# Association of the *GALNTL6* rs558129 polymorphism with muscle strength in Japanese athletes

**DOI:** 10.5114/biolsport.2025.147010

**Published:** 2025-02-04

**Authors:** Ayumu Kozuma, Eri Miyamoto-Mikami, Mika Saito, Hiroki Homma, Minoru Deguchi, Shingo Matsumoto, Ryutaro Matsumoto, Takanobu Okamoto, Koichi Nakazato, Noriyuki Fuku, Naoki Kikuchi

**Affiliations:** 1 Graduate School of Health and Sport Science, Nippon Sport Science University, Tokyo, Japan; 2 Graduate School of Health and Sports Science, Juntendo University, Chiba, Japan; 3 Faculty of Education, Ikuei University, Gumma, Japan

**Keywords:** GALNTL6, GALNT family, Genotype, Wrestlers, Isokinetic strength, Power-oriented athletes

## Abstract

The significance of GALNTL6 rs558129 polymorphism in relation to muscle strength and power performance has been reported in several studies. However, there has been no replication study in the Japanese population. Therefore, in this study, we aimed to determine the association of GALNTL6 rs558129 polymorphism with athlete status and muscle strength in Japanese athletes. In study 1, genotype frequencies were compared between 376 power-oriented athletes (male: n = 257, female: n = 119) and 1,139 controls (male: n = 448, female: n = 691). The power-oriented athletes included wrestlers (n = 146), weightlifters (n = 151) and power lifters (n = 79). In study 2, isokinetic knee extension and flexion muscle strength tests were performed in 347 athletes (male: n = 298, female: n = 49). In both studies, GALNTL6 rs558129 polymorphism was identified using the TaqMan SNP Genotyping Assay. In study 1, there were no significant differences in genotype frequencies between the power-oriented athletes and controls. However, the frequency of TT genotype was significantly higher in wrestlers compared to controls (p = 0.044). In study 2, isokinetic knee extension muscle strength was significantly higher in athletes with the T allele than in athletes with the CC genotype (p = 0.012). In addition, a significant tendency of the isokinetic knee extension muscle strength was observed in the CC, CT, and TT genotype, respectively. The results of this study suggest that the TT genotype and T allele of GALNTL6 polymorphism are associated with knee extension muscle strength in Japanese athletes and that the TT genotype is observed at a higher frequency in Japanese wrestlers. These findings are consistent with and further validate the results of previous studies.

## INTRODUCTION

Athletic performance is influenced by both environmental factors (such as training, nutrition, and sleep) and genetic factors [1]. Despite numerous studies, the genetic underpinnings of athletic performance remain inadequately understood, particularly regarding specific gene polymorphisms [2, 3].

In 2016, two genome-wide association studies (GWAS) involving a large cohort of elite endurance athletes from Germany, Finland, Canada, and USA (GENATHLETE, national and world-class level), and Japan (world-class level) aimed to identify genetic variants associated with superior endurance performance [4]. This study highlighted the significance of the N-acetylgalactosaminyltransferase-like 6 (*GALNTL6*) rs558129 polymorphism, particularly noting a higher frequency of the C allele in Japanese endurance athletes compared to Japanese controls (p = 3.01 × 10^−5^). A subsequent metaanalysis involving endurance athletes from GENATHLETE and seven countries (Australia, Ethiopia, Japan, Kenya, Poland, Russia, and Spain) confirmed these findings in multiple ethnic groups.

*GALNTL6*, a member of the mammalian GALNT family, is pivotal in initiating mucin-type O-glycosylation, a critical process in human physiology [5]. The *GALNTL6* rs558129 polymorphism is located in the gene’s last intron and *GALNTL6* encodes N-acetylgalactosaminyltransferase-like 6. Previous study reported polymorphism in the GALNT family, particularly *GALNT13*, are associated with sprint athletic status, a different phenotype from endurance performance, which has been previously reported for *GALNTL6* [6]. Therefore, the GALNT family, including *GALNTL6*, might be associated with muscle strength and power athletic performance, in addition to endurance performance.

Current studies have also focused on the associations of the *GALNTL6* rs558129 polymorphism with muscle strength and power phenotypes in European populations [7, 8]. Diaz Ramirez et al. [7] reported higher T allele frequencies in strength and power athletes compared to those observed in controls. The mean and peak power (absolute and relative) of the 30-second Wingate test is also suggested to be significantly higher for the CT+TT genotype than for the CC genotype. Moreover, Zmijewski et al. [8] observed higher frequencies of the T allele and CT + TT genotype in short-distance swimmers compared to controls. However, these results do not examine the relationship between this polymorphism and power in the Japanese population, where the association with performance was first identified.

Given the potential ethnic specificity suggested by previous studies, in this study, we aimed to investigate the association of the *GALNTL6* rs558129 polymorphism with athletic performance in Japanese power-oriented athletes. We hypothesize that this polymorphism may be linked to enhanced muscle strength and power performance in this population.

This study conducts a case-control study to examine the relationship between the *GALNTL6* rs558129 C/T polymorphism and athlete status (Study 1) and to compare the association of the *GALNTL6* rs558129 C/T polymorphism with muscle strength in Japanese athletes (Study 2), addressing the gap in the current understanding of the impact of this polymorphism on athletic phenotypes.

## MATERIALS AND METHODS

### Subjects

The total number of subjects was 1,862, all of whom were Japanese. Study 1, a case-control study involving 1,515 Japanese subjects, including 376 power-oriented athletes (257 men, 119 women, 23.6 ± 7.9 years) and 1,139 healthy Japanese controls from the living in Tokyo area (448 men, 691 women, 54.3 ± 15.2 years). The athletes participated in both national and international competitions, including Olympic games, comprising 146 wrestlers (65 international levels, 81 national levels), 151 weightlifters (55 international levels, 96 national levels), and 79 powerlifters (29 international levels, 50 national levels). The muscle strength measurements in Study 2 included 347 university athletes (298 men, 49 women, 19.7 ± 1.4 years). University athletes were required to be members of a sports club, have no lower limb injuries, and individuals with incomplete data were excluded. Subjects in Study 2 were drawn from a different population, with no individuals shared between Study 1 and Study 2. Written consent was obtained from all subjects before the study was conducted. The study was approved by the ethics committees of Juntendo University (Number: GSHSS2022-52, GSHSS2021-135) and Nippon Sport Science University (Number: 020-G03) and was conducted in accordance with the principles of the Declaration of Helsinki.

### Measurements

#### Isokinetic knee extension and flexion muscle strength tests (Study 2)

Isokinetic knee extension and flexion muscle strengths were assessed using a dynamometer (Biodex System 3 and System 4, New York, USA). The measurements were performed with the subjects in a seated position. To ensure stability during the assessment, the trunk, waist, and dominant thigh and leg were secured with belts, and additional trunk belts crossing the chest were held by the subjects with both hands. The axis of rotation was aligned with the center of the dynamometer at the lateral femoral condyle. The range of motion extended from maximum extension to maximum flexion. Gravity correction was applied with the knee joint at approximately 20 degrees of flexion while the thigh was decompressed. Subjects completed several sub-maximal repetitions with progressively increasing loads as a warm-up to familiarize themselves with the movements. For the measurements, the angular velocity was set to 60°/s, and each subject performed three trials. All subjects had prior experience with this procedure and were accustomed to the testing protocol. During the assessment, subjects were verbally encouraged by test personnel to exert maximum effort. All measurements were conducted by experienced examiners to ensure consistency and minimize variability. The maximum muscle strength of the dominant leg, normalized to each subject’s body weight, was used as the final result.

### Genotyping

Saliva samples were collected using an Oragene-DNA Kit (DNA Genotek, ON, Canada). Saliva samples were incubated at 50°C for at least 1 hour to promote cell lysis. Subsequently, a purification reagent (PT-L2P) was added to precipitate proteins and other impurities. The mixture was centrifuged, and the supernatant containing DNA was carefully transferred to a new tube. DNA was then precipitated using ethanol, followed by washing to remove residual impurities. Finally, the DNA pellet was resuspended in TE buffer or an appropriate elution buffer and stored at 4°C until further analysis. Analysis of the *GALNTL6* rs558129 polymorphism was performed using Bio-Rad PCR System (CFD-3120J1 PCR System, Bio-Rad, Hercules, CA, USA) or QuantStudio 5 Real-Time PCR System (Thermo Fisher Scientific) and identified using the TaqMan SNP Genotyping Assay (C___968950_10). Bio-Rad PCR System used 5 μL genotyping reactions consisting of 2.5 μL of TaqMan® GTXpressTM MasterMix (applied biosystems), 0.125 μL of TaqMan SNP Genotyping Assay, 2.375 μL of distilled water, and 1 μL of genomic DNA. Additionally, the QuantStudio 5 Real-Time PCR System utilized a 5 μL genotyping reaction comprising 2.5 μL of TaqMan GTXpress Master Mix (2 ×), 0.0625 μL of TaqMan SNP Genotyping Assay Mix (40 ×), 1.4375 μL of distilled water, and 1 μL of genomic DNA. The thermal cycling conditions consisted of an initial denaturation at 95°C for 20 s, followed by 40 cycles of denaturation at 95°C for 3 s and annealing/extension at 60°C for 20 s. These protocols are similar to our previous studies [9, 10].

### Statistical analysis

All statistical analyses were performed using SPSS Statistics version 25 (IBM Japan, Tokyo, Japan). Pearson’s Chi-squared test was performed to confirm the Hardy-Weinberg equilibrium in genotype frequencies and to compare allele and genotype frequencies between the power-oriented athletes and the controls and evaluate the male and female ratio among the subjects. The subject’s characteristics (age, height, body weight, and athletic experience) for each genotype were assessed using one-way analysis of variance (ANOVA). Absolute isokinetic knee extension and flexion muscle strengths are shown as absolute and relative values (muscle strength/body weight). The absolute value was analyzed using one-way analysis of covariance (ANCOVA) adjusted for age, sex and weight, while relative value was analyzed using ANCOVA adjusted for age and sex. In addition, isokinetic knee extension and flexion muscle strengths were conducted with the Jonckheere-Terpstra trend test for trend. Moreover, Linear regression analysis was used to estimate the proportion of variance attributable to *GALNTL6* rs558129 polymorphism for isokinetic knee extension and flexion muscle strength. Examinations in men and women were analyzed using ANCOVA, adjusting for age and weight in absolute values and age in relative values. Statistical significance was set at p < 0.05.

## RESULTS

### Study 1: Case-control study in power-oriented athletes

A comparison of the genotype frequencies between the power-oriented athletes and controls is shown in Table 1. The *GALNTL6* rs558129 polymorphism frequencies in the controls were in Hardy– Weinberg equilibrium (p = 0.410) and similar to those observed in the Japanese population database (https://jmorp.megabank.tohoku.ac.jp/). There were no significant differences found in genotype frequencies between power-oriented athletes and controls. However, when the athletes were grouped by sport events, the frequency of the TT genotype was found to be significantly higher in the wrestlers than in the controls (Table 1, p = 0.044).

**TABLE 1:** Genotype and allele frequencies of *GALNTL6* rs558129 polymorphism in power-oriented athletes and controls

	n	Genotype n (%)			Allele n (%)		p-value				

		CC (%)	CT (%)	TT (%)	Allele C (%)	Allele T (%)	Genotype	Additive	Allele	CC vs CT+TT	CC+CT vs TT
All power athletes	376	264 (70)	95 (25)	17 (5)	623 (83)	129 (17)	0.336	0.586	0.578	0.941	0.152
Wrestlers	146	101 (69)	36 (25)	9 (6)	238 (82)	54 (18)	0.127	0.349	0.339	0.758	0.044
Weightlifters	151	108 (72)	38 (25)	5 (3)	254 (84)	48 (16)	0.916	0.864	0.862	0.778	0.825
Power lifters	79	55 (72)	21 (25)	3 (3)	131 (83)	27 (17)	0.919	0.794	0.791	0.881	0.684
Controls	1139	802 (70)	303 (27)	34 (3)	1907 (84)	371 (16)	-	-	-	-	-

In addition, this data was compared by sex. No significant differences were found between power-oriented athletes and controls in either male or female (Supplementary table 1).

### Study 2: Muscle strength in athletes

**FIG. 1:**
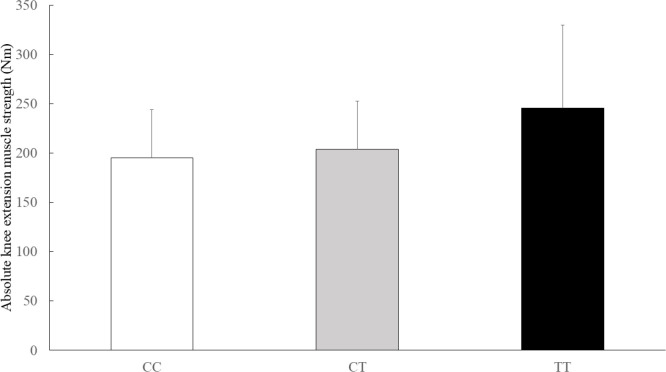
Comparison of the absolute isokinetic knee extension muscle strength by genotype Data are presented as mean ± SD. p = 0.029 (adjusted for age, sex and weight by ANCOVA), p = 0.034 (Jonckheere-Terpstra trend test)

**FIG. 2:**
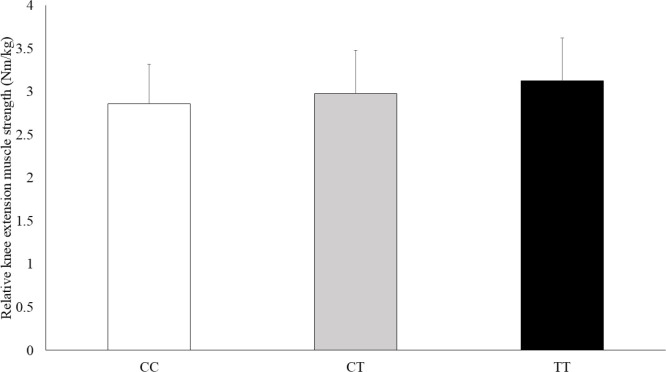
Comparison of the relative isokinetic knee extension muscle strength by genotype. Data are presented as mean ± SD. p = 0.029 (adjusted for age and sex by ANCOVA), p = 0.053 (Jonckheere-Terpstra trend test)

The physical characteristics of the athletes by *GALNTL6* rs558129 polymorphisms are shown in Table 2. The absolute and relative isokinetic knee extension muscle strength values were significantly different between genotype and C recessive model (Table 3). The C recessive model for the *GALNTL6* rs558129 polymorphism accounted for 1.1% of the variability in the absolute values and 1.4% of the variability in the relative values of isokinetic knee extension muscle strength. In addition, Jonckheere-Terpstra trend test showed that the absolute and relative isokinetic knee extension muscle strength values were higher in the order of CC, CT, and TT genotype (Figure 1, absolute: p = 0.034, Figure 2, relative: p = 0.053). However, there were no significant differences in absolute and relative isokinetic knee flexion muscle strength values.

**TABLE 2:** Results of characteristics by *GALNTL6* rs558129 polymorphism in athletes

Characteristics	Genotype			

	CC (n = 238)	CT (n = 98)	TT (n = 11)	p-value
Male / Female	204 / 34	84 / 14	10 / 1	0.888
Age (years)	19.7 ± 1.4	19.5 ± 1.2	20.8 ± 1.3	0.026
Height (cm)	171.9 ± 7.9	172.0 ± 7.8	176.7 ± 5.4	0.142
Weight (kg)	67.8 ± 11.2	68.2 ± 10.1	77.4 ± 21.1	0.027

Data are presented as mean ± SD.

**TABLE 3:** Results of muscle strength by *GALNTL6* rs558129 polymorphism in athletes

	Genotype				C dominant		C recessive	

	CC (n = 238)	CT (n = 98)	TT (n = 11)	p-value (Genotype)	CC + CT (n = 336)	p-value (C dominant)	CT + TT (n = 109)	p-value (C recessive)
Absolute knee extension (Nm)*	195.2 ± 48.9	203.9 ± 48.7	245.6 ± 84.4	0.029	197.7 ± 48.9	0.134	208.1 ± 54.3	0.012

Relative knee extension (Nm/kg)†	2.8 ± 0.4	2.9 ± 0.4	3.1 ± 0.4	0.029	2.8 ± 0.4	0.191	2.9 ± 0.4	0.010

Absolute knee flexion (Nm)*	103.4 ± 25.7	106.6 ± 25.5	117.8 ± 48.8	0.282	104.3 ± 25.6	0.447	107.8 ± 28.5	0.252

Relative knee flexion (Nm/kg)†	1.5 ± 0.2	1.5 ± 0.2	1.4 ± 0.3	0.206	1.5 ± 0.2	0.305	1.5 ± 0.2	0.264

Data are presented as mean ± SD.

* adjusted for age, sex and weight by ANCOVA.

† adjusted for age and sex by ANCOVA.

When the athletes were grouped by sex, genotype-related differences in isokinetic knee extension muscle strength (absolute and relative) were observed among the male athletes (Supplementary table 2, absolute: p = 0.046, relative: p = 0.036).

## DISCUSSION

This study examined the association of the *GALNTL6* rs558129 polymorphism with muscle strength and athlete status in a Japanese cohort. The results of Study 1 indicated no significant differences in genotype and allele frequencies between power-oriented athletes (weightlifters, powerlifters, wrestlers) and controls, but the frequency of the TT genotype was significantly higher in wrestlers. In Study 2, isokinetic knee extension muscle strength was significantly higher in individuals with the CT + TT genotype compared to other genotypes. Furthermore, Jonckheere-Terpstra trend test showed an increase in muscle strength from the CC, CT and TT genotype, respectively.

Previous studies have reported varying associations between the *GALNTL6* rs558129 polymorphism and athletic performance, C allele is associated with endurance performance and T allele is associated with muscle strength and power performance [4, 7, 8]. Diaz Ramirez et al. found that the CT + TT genotype was associated with higher mean and peak power in the 30 s Wingate anaerobic test, similar to our findings regarding muscle strength in athletes [7]. Additionally, their case-control study revealed a higher frequency of the CT + TT genotype in strength and power Olympic-level athletes compared to that in controls. Similarly, Zmijewski et al. reported higher frequencies of the T allele and CT + TT genotype in short-distance swimmers [8]. Our study found no significant differences in power-oriented athletes overall; however, when stratified by sport, wrestlers had a significantly higher frequency of the TT genotype. The results of this study may be influenced by the higher proportion of wrestlers who were Olympic and international competitors compared to weightlifters and powerlifters at the competition level of the subjects.

The *GALNTL6* gene is highly expressed in the testis [11], where it may play a role in spermatogenesis and potentially influence sex hormone levels, such as testosterone. Testosterone plays a crucial role in muscle strength [12]. Previous studies have demonstrated an association between testosterone levels and muscle strength or power in athletes [13, 14]. Although the direct relationship between *GALNTL6* and testosterone has not yet been explored, another member of the GALNT family, *GALNT13*, has been reported to be associated with free testosterone levels [15]. In our study, significant differences in muscle strength were observed among athletes. These findings suggest that *GALNTL6* may influence muscle function through its potential impact on testosterone. However, further studies are required to clarify this mechanism, and sex differences should be investigated in greater detail in future research.

Our results also align with broader genetic research indicating associations between various *GALNT* polymorphisms and traits related to athletic performance. The GWAS catalog includes 36 studies on *GALNTL6* polymorphisms, highlighting their relevance in areas such as body size, memory, education and smoking habits [16–20]. Although no gene polymorphisms are in complete linkage disequilibrium, the *GALNTL6* polymorphism may be associated with athletic performance. Moreover, polymorphisms in other GALNT family genes, like *GALNT13*, have been associated with sprint performance, testosterone levels, reaction time, and body size, suggesting a broader role for this gene family in physical capabilities [6, 16, 17, 21–23].

The limitations of this study include the age difference between athletes and controls and the small sample size of female athletes, which may have led to residual confounding despite adjustments for age and related factors. Additionally, muscle strength measurements were conducted only at an angular velocity of 60°/s, leaving uncertainty about whether similar results would be observed at other velocities. Another limitation is the lack of consideration for muscle mass and biochemical markers, such as testosterone levels, which are crucial factors influencing muscle strength and power. Moreover, although this study focused on a single gene polymorphism, muscle strength is influenced by a combination of genetic and environmental factors, such as training, nutrition, and epigenetic modifications. Notably, the contribution of the *GALNTL6* rs558129 polymorphism to muscle strength was limited (1.1–1.4%), underscoring the multifactorial nature of muscle function. Future studies should take these factors into account to provide a more comprehensive understanding. Additionally, although this study primarily examined single gene polymorphisms, future genomics research is essential to further explore the complex relationship between genetic factors and athletic performance [24, 25].

## CONCLUSIONS

In conclusion, our findings suggest that the TT genotype and CT + TT genotype of the *GALNTL6* rs558129 C/T polymorphism are associated with enhanced muscle strength in Japanese athletes. In addition, TT genotype in *GALNTL6* rs558129 C/T polymorphism might be associated with athletic status in wrestlers. These findings are consistent with and further validate the results of previous studies.

Further studies are needed to explore the physiological mechanisms underlying this association and to confirm these findings in larger and more diverse populations. Incorporating subjects from various ethnic backgrounds will also be essential to evaluate potential ethnic differences in genetic associations.

## Supplementary Material

Association of the *GALNTL6* rs558129 polymorphism with muscle strength in Japanese athletes
