# Supplementary material for: Association of the GALNTL6 rs558129 polymorphism with muscle strength in Japanese athletes
Source: Biol Sport. 2025 Feb 4;42(3):161–7. doi: 10.5114/biolsport.2025.147010 (PMC12314483; doi:10.5114/biolsport.2025.147010)
Supplement: Association of the GALNTL6 rs558129 polymorphism with muscle strength in Japanese athletes [file JBS-42-3-55558-s1.pdf]

SUPPLEMENTARY TABLE 1. Genotype and allele frequencies of *GALNTL6* rs558129 polymorphism in power-oriented athletes and controls by sex

	n	Genotype n (%)			Allele		p-value				
		**CC (%)**	**CT (%)**	**TT (%)**	**C (%)**	**T (%)**	**Geno-type**	**Addi-tive**	**Allele**	**CC vs CT+TT**	**CC+CT vs TT**
Male athletes	257	172 (67)	71 (28)	14 (5)	415 (81)	99 (19)	0.391	0.176	0.157	0.234	0.302
Female athletes	119	92 (77)	24 (20)	3 (3)	208 (87)	30 (13)	0.231	0.15	0.15	0.099	0.968
Male controls	448	319 (71)	112 (25)	17 (4)	750 (84)	146 (16)					
Female controls	691	483 (70)	191 (28)	17 (2)	1157 (84)	225 (16)					

SUPPLEMENTARY TABLE 2. Results of muscle strength by *GALNTL6* rs558129 polymorphism in male and female athletes

	**Male**				**Female**			
	**CC (n = 204)**	**CT (n = 84)**	**TT (n = 10)**	**p-value**	**CC (n = 34)**	**CT (n = 14)**	**TT (n = 1)**	**p-value**
Absolute knee extension (Nm)*	203.4 ± 45.5	210.3 ± 46.2	257.0 ± 79.6	0.046	146.2 ± 39.3	165.1 ± 46.5	132.4	0.902
Relative knee extension (Nm/kg)†	2.9 ± 0.4	3.0 ± 0.4	3.1 ± 0.4	0.036	2.4 ± 0.4	2.5 ± 0.4	2.4	0.635
Absolute knee flexion (Nm)*	107.5 ± 24.8	110.5 ± 24.4	122.6 ± 48.6	0.200	79.1 ± 16.5	83.4 ± 18.9	69.7	0.774
Relative knee flexion (Nm/kg)†	1.5 ± 0.2	1.6 ± 0.2	1.5 ± 0.3	0.169	1.3 ± 0.1	1.2 ± 0.1	1.2	0.908

Data are presented as mean ± SD. *adjusted for age and weight by ANCOVA. †adjusted for age by ANCOVA.

Articles published in the Biology of Sport are licensed under an open access Creative Commons CC BY 4.0 license.
